# 1386. Seroprevalence of Strongyloidiasis in Liver Transplant Candidates at a Tertiary-Level Hospital in Newark, NJ

**DOI:** 10.1093/ofid/ofab466.1578

**Published:** 2021-12-04

**Authors:** Jorge Robledo, Michael O’Shaughnessy, Tilly Varughese, Diana Finkel

**Affiliations:** 1 Rutgers New Jersey Medical School, Newark, New Jersey; 2 NJMS Rutgers University, Newark, NJ

## Abstract

**Background:**

The liver transplant center at University Hospital (Newark, NJ) is one of the busiest in northern NJ. Current guidelines for Strongyloides stercoralis (Ss) screening in solid transplant recipients recommend targeted testing. We propose a high seroprevalence of this infection in our facility given its significant percentage of foreign-born patients from Ss endemic areas such as Latin America, the Caribbean, and Africa.

**Methods:**

Descriptive study from secondary data. We obtained the total number of Strongyloides antibody tests performed at University Hospital in the last two years (08/2018-10/2020). Subsequently, medical charts were reviewed to obtain epidemiological and clinical data.

**Results:**

A total of 388 patients underwent screening for Strongyloides antibody, of whom 71 (18%) were positive. The test was mainly performed in male (58%) and foreign-born (55%) patients. More than half (55%) of the US-born individuals had history of travel overseas. The main reasons for testing were transplant evaluation (65%), immunosuppression (14%) and eosinophilia (9%). There was no association between transplant evaluation and seropositivity (81% vs 81%, p = 0.994). Being foreign-born was not associated with a positive test (19% vs 20%, p = 0.834), but for US-born patients, having a history of travel was associated with a positive test (33% vs 14%, p = 0.039). For the Ss positive patients, 34% had a HTLV-I/II test, 48% had at least one stool test, and 76% were given treatment.

**Conclusion:**

There is a significant seroprevalence of Ss in our transplant candidate population, both non-foreign and foreign-born, prompting the indication for universal screening at our facility.

**Disclosures:**

**All Authors**: No reported disclosures

